# Alternative promotion and suppression of metastasis by JNK2 governed by its phosphorylation

**DOI:** 10.18632/oncotarget.17507

**Published:** 2017-04-28

**Authors:** Sike Hu, Xiaoli Dong, Wenjuan Gao, Dwayne Stupack, Yanhua Liu, Rong Xiang, Na Li

**Affiliations:** ^1^ School of Medicine, Nankai University, Tianjin 300071, China; ^2^ Department of Reproductive Medicine, Division of Gynecologic Oncology, Moores Cancer Center, University of California San Diego, La Jolla, CA 92093-0987, United States; ^3^ Tianjin Key Laboratory of Tumour Microenvironment and Neurovascular Regulation, Tianjin 300071, China; ^4^ Collaborative Innovation Center for Biotherapy, Nankai University, Tianjin 300071, China

**Keywords:** JNK2, p-JNK2, Fra1, EMT

## Abstract

Fos-related antigen 1 (Fra1) has been proposed as a gatekeeper of the mesenchymal-epithelial transition to epithelial-mesenchymal transition. Here, we showed that de-phosphorylated JNK2 increased the expression of Fra1 by promoting the expression of c-Jun and Jun-B. Conversely, phosphorylated JNK2 suppressed its expression via enhancing the ubiquitination of c-Jun and Jun-B. These data provided insights into the regulatory mechanism of JNK2 on the expression of Fra1. Our study thus demonstrated that the conversion of JNK2 from its phosphorylation to de-phosphorylation status promoted the switch of breast cancer cells from mesenchymal-epithelial transition to epithelial-mesenchymal transition.

## INTRODUCTION

Epithelial-mesenchymal transition (EMT) is an important metastasis-associated process, in which cancer cells obtained the metastatic competence of migratory and invasive capabilities [1]. However, the molecular mechanisms regulating this complex process still need to be explored. A better understanding of this process is essential to develop new therapeutic strategies for cancer.

The c-Jun N-terminal kinases (JNKs) can be activated by a range of stimuli and were labeled as “stress-activated protein kinases”. JNKs include JNK1, JNK2 and JNK3. They are encoded by distinct genes [2–4]. Each of them has multiple isoforms, at least 4 isoforms of JNK1, 4 isoforms of JNK2 and 2 isoforms of JNK3 could be generated through alternative splicing of their pre-mRNAs. Among them, JNK1α1, JNK1β1, JNK2α1, and JNK2β1 are 46 kDa while JNK1α2, JNK1β2, JNK2α2, and JNK2β2 are 54 kDa. All of 10 isoforms have the same phosphorylation site [2–7]. JNK1 and JNK2 were disclosed to be expressed ubiquitously whereas JNK3 was reported to be expressed predominantly in the brain, testis and heart tissues [7–10]. JNK1 and JNK2 play important roles in tumor metastasis. JNK1 phosphorylated multiple proteins, such as microtubule associated proteins 2 and 1B, paxillin, and stathmin-2, to influence or enhance cell motility [11–14]. JNK2 inhibited the expression of epidermal growth factor receptor pathway substrate 8 (EPS8), which controlled the actin-based motility and were involved in regulating Rac-mediated cell migration, to promote the migration of mammary tumor cells [15–18]. JNK2-selective peptide inhibitors were reported to attenuate the migration of breast cancer cells [19]. In addition, the metastasis-promotion effect of JNK2 was found to be dependent on the molecular phenotype of breast tumors. JNK2 expression did not affect the disease free survival (DFS) for the patients with all subtypes of breast tumors. However, high expression level of JNK2 was associated with poorer DFS in patients with basal-type tumors [16].

Fos-related antigen 1 (Fra1) is an effective regulator of invasion and migration in a variety of solid tumors. The expression of Fra1 was shown to be correlated with the mesenchymal characteristics of epithelial tumors. Overexpression of Fra1 in epithelioid adenocarcinoma cells greatly influenced cell motility, morphology and invasiveness [20]. Fra1 was regarded as the gatekeeper for the mesenchymal-epithelial transition (MET) to EMT in breast cancer [21] and directly control the epithelial-mesenchymal plasticity in colorectal cancer (CRC) cells [22].

To date, for the MAPKs pathway signaling, only ERK1/2 signaling pathway was found to be critical for EMT by recruiting c-Jun at the *Fra1* promoter [23]. So far, no signaling pathway was reported to inhibit the expression of Fra1 according to our knowledge. In this paper, we investigated the correlation between JNK2 and Fra1 in different breast cancer cells. We found that de-phosphorylation of JNK2 promoted, but phosphorylation of JNK2 inhibited the expression of Fra1 as well as cell mobility in a c-Jun, Jun-B dependent manner. This study thus disclosed vital function of JNK2 in regulating the MET to EMT procedure in breast cancer cells.

## RESULTS

### JNK phosphorylation inversely correlated with Fra1-protein levels in breast cancer cells

**Figure 1:**
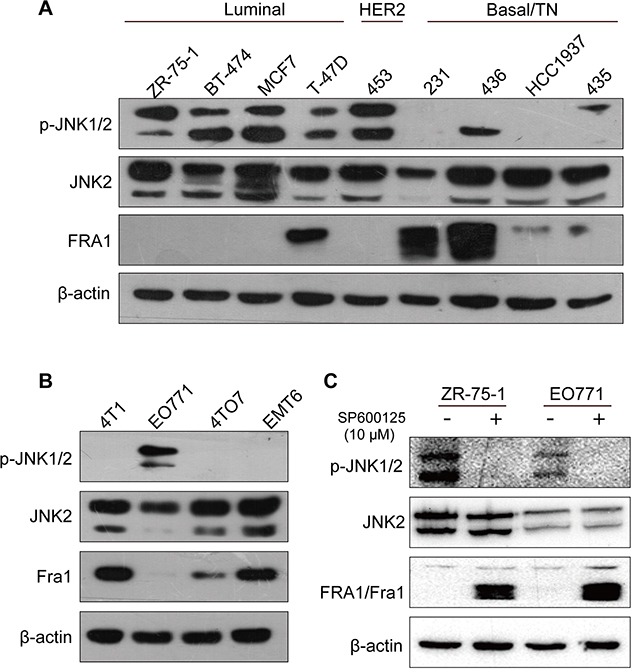
Fra1 level inversely correlated with the phosphorylation status of JNK in different types of breast cancer cell lines **(A-B)** Western blotting result showing the expression of p-JNK, JNK2 and FRA1 in a panel of human and breast cancer cell lines. 453, 231, 436 and 435 stand for MDA-MB-453, MDA-MB-231, MDA-MB-436 and MDA-MB-435 separately. **(C)** The expression of p-JNK, JNK2 and FRA1/Fra1 was detected by Western blot in EO771 and ZR-75-1 cells after treatment with JNK inhibitors SP600125 and DMSO control for 24h.

To test the possible functional correlation between JNK2 and Fra1, expressions of these two proteins were detected in a panel of human breast cancer cell lines. No inherent differences for the expression of JNK2 were found in these breast cancer cells with luminal, HER2 or basal /triple negative (TN) phenotype (Figure 1A). Except in T-47D, FRA1 was absent/very low expressed in other 4 human breast cancer cell lines with luminal or HER2 phenotype, in which p-JNK1/2 was overexpressed. In contrast, expression of FRA1 was detected in 4 breast cancer cell lines with basal /TN phenotype, in which the expression level of p-JNK1/2 was low (Figure 1A). These results suggested that p-JNK1/2 may inhibit the expression of FRA1. To determine if the expression of Fra1 might generally correlate with the absence of p-JNK1/2, we extended these studies to murine breast cancer cell line (Figure 1B). Interestingly, Fra1 was expressed in all the murine cells lacking phosphorylation of JNK1/2 (4T1, 4TO7 and EMT6), but was very low expressed in EO771 cells which showed high expression level of p-JNK1/2 (Figure 1B).

To investigate this underlying mechanism, human ZR-75-1 cells and mouse EO771 cells, which showed relative high levels of p-JNK1/2, were incubated with the JNK inhibitor SP600125. As shown in Figure 1C, transient treatment with the JNK inhibitor resulted in loss of p-JNK1/2 and the *de novo* induction of FRA1/Fra1 expression. Together, the data raised a compelling possibility: the expression of Fra1 may be inhibited by p-JNK1/2.

### JNK2-suppression had contrasting effects in different breast cancer cell lines

**Figure 2:**
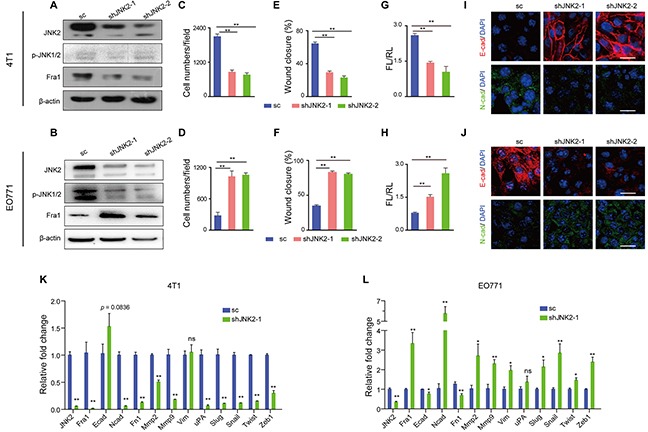
Down-regulation of *JNK2* had different effects on cell migration in mouse breast cancer cells **(A-B)** Western blot analysis of JNK2, p-JNK and Fra1 in 4T1 cells (A) and EO771 cells (B) infected with lentivirus carrying shRNAs targeting JNK2 or a non-targeting scramble control (sc). **(C-D)** Transwell assay results of 4T1 cells (C) and EO771 cells (D) with or without *JNK2* down-regulation (n = 5 for 4T1, n = 3 for EO771). **(E-F)** Wound healing assay results of 4T1 cells (E) and EO771 cells (F) with or without *JNK2* down-regulation (n = 8 for 4T1, n = 10 for EO771). **(G-H)** Effect of JNK2 on *Fra1*-reporter luciferase activity was shown in 4T1 (G, n = 3) and EO771 (H, n = 3-6). **(I-J)** Immunofluorescent staining of E-cadherin (E-cad, red), N-cadherin (N-cad, green) in 4T1 (I) and EO771 cells (J) with or without *JNK2* down-regulation. The nuclei were counterstained with DAPI (blue). Scale bar: 50 μm. **(K-L)** Real-time quantitative RT-PCR for mRNAs expression changes of genes associated with EMT and metastasis in 4T1 cells (K) and EO771 cells (L) following *JNK2* knockdown (n = 3).

To investigate the two subtypes of JNK in regulating the expression of Fra1, small hairpin RNAs (shRNAs) targeting JNK1 and JNK2 were introduced into 4T1 cells (TN phenotype), which showed relative high expression of JNK2, but absence/ very low expression level of p-JNK1/2, and into EO771 cells (basal-like phenotype), which expressed both JNK2 and p-JNK1/2. We found that in both cell lines, knockdown of JNK1 had no effect on the mRNA expression level of *Fra1* (Supplementary Figure 1A, 1B). But in 4T1, silencing of *JNK2* decreased the expression of Fra1 (Figure 2A), accompanied by significantly reduced migration ability (Figure 2C, 2E, Supplementary Figure 1C). Concordant with inhibition, there was a decreased expression of mesenchymal marker N-cadherin (N-cad) and Cytokeratin 14 (CK14) as detected by immunofluorescence (Figure 2I, Supplementary Figure 1E). In parallel, the expressions of epithelial markers E-cadherin (E-cad) and Cytokeratin 8 (CK8) were elevated (Figure 2I, Supplementary Figure 1E).

However, in EO771 cells, the results were completely reversed for each case. Knockdown of *JNK2* endowed cells with mesenchymal traits as well as high mobility property, which was accompanied by gained expression of Fra1 and lost expression of epithelial markers. These cells were shown to possess more malignant phenotype (Figure 2B, 2D, 2F, 2J, Supplementary Figure 1D, 1F).

Correspondingly, the acute loss of JNK2 in 4T1 cells occurred concordantly with suppressed *Fra1-*promoter activity (Figure 2G) and with downregulated mRNA expression of many mesenchymal-and-metastasis-related genes as well as *Fra1* (Figure 2K). By contrast, *Fra1-promoter* activity increased in the EO771 cells (Figure 2H), concordant with a generally increased mRNA expression of mesenchymal-and-metastasis-related marker genes as well as *Fra1* (Figure 2L). In addition, when we depleted JNK2 in the two human breast cancer cells MDA-MB-231, which showed absent/very low expression level of p-JNKs and in MDA-MB-453, which expressed both JNK2 and p-JNKs, distinct loss or gain of FRA1- expression were also observed in these two cell lines (Supplementary Figure 1G).

Together, these results demonstrated that loss of JNK2 showed distinct effects on the EMT and migration properties in different breast cancer cells. They also suggested that phosphorylation of p-JNK2 may inhibit the expression of Fra1, whereas de-phosphorylated JNK2 may promote its expression.

### Expression of constitutively active mitogen-activated protein kinase kinase 7 (MKK7) resulted in JNKs phosphorylation, reduced expression of Fra1 and inhibited breast cancer mobility

**Figure 3:**
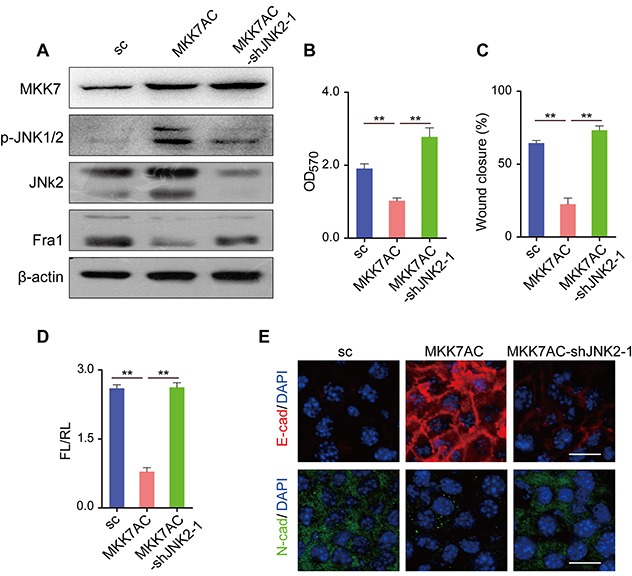
Overexpression of *MKK7AC* to promote the phosphorylation of JNK2 inhibited the metastasis of 4T1 cells **(A)** Western blot results of MKK7, p-JNK, JNK2 and Fra1 in 4T1-sc, 4T1-MKK7AC and 4T1-MKK7AC-shJNK2-1 cells. **(B)** Transwell assay results of 4T1-sc, 4T1-MKK7AC and 4T1-MKK7AC-shJNK2-1 cells (n = 3). **(C)** Wound healing assay results of 4T1-sc, 4T1-MKK7AC and 4T1-MKK7AC-shJNK2-1 cells (n = 8). **(D)** *Fra1*-reporter luciferase activity in 4T1-sc, 4T1-MKK7AC and 4T1-MKK7AC-shJNK2-1 cells, n = 3. **(E)** Immunofluorescent staining of E-cad (red), N-cad (green) in 4T1-sc, 4T1-MKK7AC and 4T1-MKK7AC-shJNK2-1 cells, the nuclei were counterstained with DAPI (blue). Scale bar: 50 μm.

MKK7 and MKK4 work as crucial upstream transducers of JNK signaling. Once activated, MKK7 and MKK4 directly phosphorylate specific tyrosine and threonine residues located in the conserved Thr-X-Tyr motif of the phosphorylation/activation loop of the JNK proteins [3]. To further test the possibility that p-JNK2 inhibits the expression of Fra1, we expressed constitutively active MKK7 (MKK7AC), thus promoting phosphorylation of JNKs in 4T1 cells. We found overexpression of *MKK7AC* in 4T1 cells (4T1-MKK7AC) resulted in the acquisition of p-JNKs, together with reduced Fra1-expression (Figure 3A) and a concomitant compromised cell migration in the transwell and wound healing assays when compared to 4T1-sc (Figure 3B, 3C and Supplementary Figure 2A). However, silencing of *JNK2* in the 4T1-MKK7AC cells (4T1-MKK7AC-shJNK2-1) decreased the expression level of p-JNKs and rescued the expression of Fra1. The migration ability of the cells also recovered correspondingly when compared with 4T1-MKK7AC cells (Figure 3A, 3B, 3C and Supplementary Figure 2A). Simultaneously, *Fra1* promoter activity and the expression of EMT markers tracked with the migration results (Figure 3D, 3E and Supplementary Figure 2D). We also overexpressed *MKK7AC* in human breast cancer cell MDA-MB-231, in which the p-JNKs were absent/very low expressed, and the similar results were obtained (Supplementary Figure 2B, 2C, 2E). Similarly, compared to 4T1-sc, the expression of Fra1 was reduced when *MKK4AC* was overexpressed in 4T1 cells (4T1-MKK4AC) to induce the phosphorylation of JNKs. Its expression also recovered when shJNK2 was introduced and the expression of p-JNK2 decreased in 4T1-MKK4AC-shJNK2-1 as compared to 4T1-MKK4AC (Supplementary Figure 2F). These results supported the notion that p-JNK2 was one of the vital factors that suppressed the expression of Fra1 in breast cancer cells.

### Silencing of mitogen-activated protein kinase kinase kinase 1 (*MEKK1*) promoted metastasis and increased the expression of Fra1 in breast cancer cells

**Figure 4:**
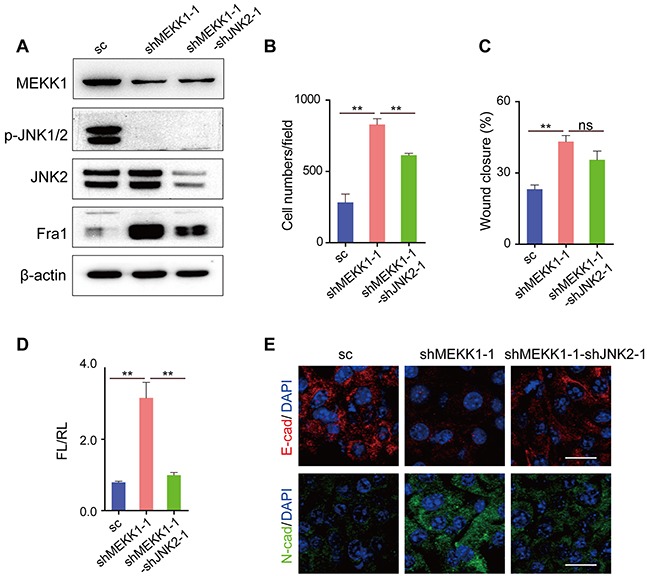
Silencing of *MEKK1* to inhibit the phosphorylation of JNK2 promoted the metastasis of EO771 cells **(A)** Western blot result of MEKK1, p-JNKs, JNK2 and Fra1 in cells of EO771-sc, EO771-shMEKK1-1 and EO771-shMEKK1-1-shJNK2-1. **(B)** Transwell assay results of EO771-sc, EO771-shMEKK1-1 and EO771-shMEKK1-1-shJNK2-1 cells (n = 3). **(C)** Wound healing assay results of EO771-sc, EO771-shMEKK1-1 and EO771-shMEKK1-1-shJNK2-1 cells (n = 7). **(D)** *Fra1* reporter luciferase activity was shown in cells of EO771-sc, EO771-shMEKK1-1 and EO771-shMEKK1-1-shJNK2-1 (n = 3-6). **(E)** Immunofluorescent staining of E-cad (red), N-cad (green) in EO771-shMEKK1-1 and EO771-shMEKK1-1-shJNK2-1 cells. The nuclei were counterstained with DAPI (blue). Scale bar: 50 μm.

MEKK1 is a protein kinase of the STE11 family, phosphorylates and activates MKK4 and MKK7, which in turn activate JNK1, JNK2 and JNK3 [3]. To explore the function of de-phosphorylated JNK2 in the expression regulation of Fra1, we used EO771 cells, in which p-JNKs were highly expressed and Fra1 was slightly expressed. We suppressed the expression of the *MEKK1* by two shRNAs in EO771 cells (EO771-shMEKK1). As shown in Figure 4A and Supplementary Figure 3E, upon *MEKK1*-silencing, inhibited phosphorylation of JNKs and acquisition of Fra1-expression were observed in EO771-shMEKK1 as compared to the EO771-sc. This was similar to the expression changes of these two proteins observed in EO771 treated with the JNKs inhibitor (Figure 1C). However, silencing of *JNK2* in EO771-shMEKK1 cells (EO771-shMEKK1-shJNK2) decreased the expression of Fra1 when compared to EO771-shMEKK1 (Figure 4A, Supplementary Figure 3E). Notably, the induction of Fra1-expression appeared to be dependent upon the expression of JNK2. The enhancement of cell migration was confirmed by the transwell and wound healing assays in EO771-shMEKK1-1 when compared with EO771-sc. However, migration was inhibited in EO771-shMEKK1-1- shJNK2-1 when compared to EO771-shMEKK1 (Figure 4B and 4C, Supplementary Figure 3A).

*Fra1* promoter activity and the expression of EMT markers tracked with the migration results (Figure 4D, 4E, Supplementary Figure 3D). Specifically, faster migration was associated with the relative high expression of CK14 and N-cad, while the cells showed slower migration properties expressed high level of CK8 and E-cad (Figure 4E and Supplementary Figure 3D). Similarly, when we silenced the expression of *MEKK1* in human breast cancer cell MDA-MB-453 (MDA-MB-453-shMEKK1-1), in which both JNK2 and p-JNKs were presented, down-regulation of p-JNKs, increased expression of FRA1 and enhanced cell migration were found (Supplementary Figure 3B, 3C, 3F). Furthermore, knockdown of *JNK2* in MDA-MB-453-shMEKK1-1 cells (MDA-MB-453-shMEKK1-1-shJNK2-1) decreased the expression of FRA1 and compromised the cell-mobility (Supplementary Figure 3B, 3C, 3F) as compared to MDA-MB-453-shMEKK1-1. Those results above supported the notion that loss of MEKK1 promoted the expression of Fra1 in breast cancer and further supported the concept that p-JNK2 inhibited the expression of Fra1.

### JNK2 regulated the transcript of *Fra1* via c-Jun and Jun-B

**Figure 5:**
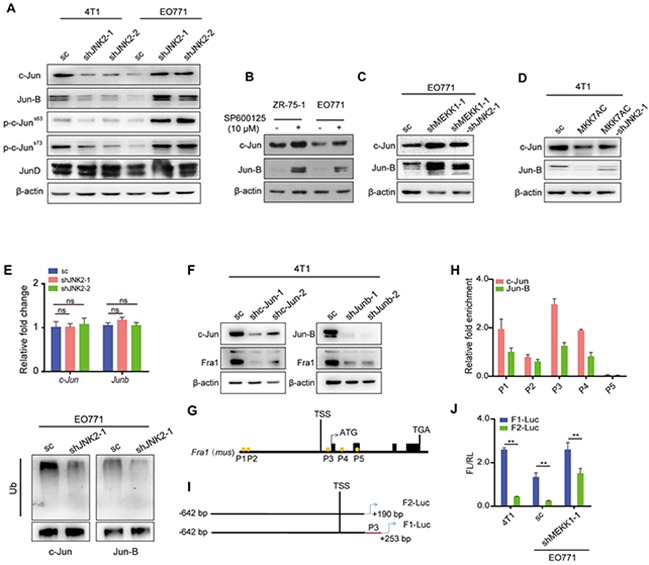
JNK2 regulated the transcript of *Fra1* through c-Jun and Jun-B, which bound to the promoter of *Fra1* **(A)** Western blot of c-Jun, Jun-B, p-c-Jun (Ser63), p-c-Jun (Ser73), and JunD in 4T1 and EO771 cells with or without *JNK2* silencing (A). **(B-D)** Western blot of c-Jun and Jun-B in the cells below: EO771 and ZR-75-1 cells treated with SP600125 and DMSO control for 24h (B); EO771-sc, EO771-shMEKK1-1 and EO771-shMEKK1-1-shJNK2-1 (C) ; 4T1-sc, 4T1-MKK7AC and 4T1-MKK7AC-shJNK2-1 (D). **(E)** The mRNA expression changes of *c-Jun* and *Junb* were examined in EO771-sc and EO771-shJNK2 cells by real-time quantitative RT-PCR (upper panel, n = 3). The ubiquitination of c-Jun and Jun-B were examined through co-IP in EO771-sc and EO771-shJNK2-1 cells (lower panel). **(F)** Western blot of c-Jun, Jun-B and Fra1 in 4T1 with *c-Jun* or *Junb* down-regulation and their control cells. **(G)** The visual representation of predicted c-Jun-and-Jun-B targets on *Fra1* promoter region (P1: -17.732 kb, P2: -17.648 kb, P3: 199 bp, P4: 1157 bp and P5: 2597 bp). Five pairs of primer were designed for quantitative-PCR (Q-PCR) to detect the binding activity. **(H)** ChIP and Q-PCR analysis were performed in 4T1-wild type (wt) with anti-c-Jun and anti-Jun-B antibody. Binding enrichment was normalized to the input DNA (n = 3). **(I)** Fragments of *Fra1* promoter with or without P3 position were constructed and cloned into the PGL3 basic vector and were named as F1-Luc and F2-Luc separately. **(J)** Statistical results for the promoter activity of F1-Luc and F2-Luc in 4T1-wt, EO771-sc, EO771-shMEKK1-1, n = 3 for 4T1, n = 6 for EO771.

JUN family are critical downstream mediators of JNK signaling. We observed the consistent expression-changes of c-Jun, Jun-B and the phosphorylation forms of c-Jun with those of Fra1 following JNK2 depletion in both 4T1 and EO771 cells (Figure 5A). This indicated that the expression level of c-Jun and Jun-B was JNK2 dependent. Whereas the expression of JunD was not regulated by JNK2 in these two cell lines (Figure 5A). Exposure to the JNK inhibitor SP600125 resulted in decreased phosphorylation of JNKs, increased expression of c-Jun and Jun-B as well as increased Fra1 in both EO771 and ZR-75-1 cells (Figures 1C and 5B). Furthermore, correlated expression of Fra1 with that of c-Jun, Jun-B was also detected in EO771-shMEKK1-1 and EO771- shMEKK1-1-shJNK2-1 (Figures 5C and 4A) as well as in MDA-MB-453-shMEKK1-1 and MDA-MB-453-shMEKK1-1-shJNK2-1 (Supplementary Figure 3F). This phenomenon was also observed in 4T1-MKK7AC and 4T1-MKK7AC-shJNK2-1 (Figure 5D and 3A) as well as in MDA-MB-231-MKK7AC and MDA-MB-231-MKK7AC-shJNK2-1 (Supplementary Figure 2E). Based on these observations, we deduced that p-JNK2 may decrease the expression of Fra1 via c-Jun and Jun-B.

We found that although the mRNA expression levels of *c-Jun* and *Junb* did not change significantly in EO771-shJNK2 when compared with those in EO771-sc cells (Figure 5E), their protein expression levels increased (Figure 5A). These finding indicated that JNK2 regulated the expression of c-Jun and Jun-B through a post-transcriptional regulation method. It was reported that c-Jun and Jun-B turnover was controlled through JNK-dependent phosphorylation of the E3 ligase-Itch [24]. We observed that depletion of JNK2 in EO771 cells decreased polyubiquitination of both c-Jun and Jun-B (Figure 5E). From these data, we concluded that p-JNK2 decreased the expression of both c-Jun and Jun-B by promoting their ubiquitination.

To determine whether c-Jun and Jun-B promote the expression of Fra1 in breast cancer cells, we suppressed their expression by shRNA separately in 4T1. In fact, knockdown of either of them compromised the expression of Fra1 (Figure 5F). Five positions containing the binding motif (5′-TGA(G/C)TCA-3′) of c-Jun and Jun-B were found in the *Fra1* promoter region (Figure 5G). Quantitative-chromatin immunoprecipitation (Q-ChIP) was performed to find the enriched binding sites of c-Jun and Jun-B. The results showed that both of them were enriched at the P3 position, which was closed to the transcription start site (TSS) of *Fra1* (Figure 5H). To further disclose the regulatory mechanism of c-Jun and Jun-B on the transcription of *Fra1*, a firefly luciferase reporter containing the P3 sequence of *Fra1* gene promoter (named as F1-Luc), as well as a P3-depleted *Fra1* gene promoter (named as F2-Luc, Figure 5I) was constructed. F2-Luc abrogated luciferase activity when compared with F1-Luc in all 4T1, EO771-sc and EO771-shMEKK1-1 cells (Figure 5J). These observations demonstrated that *Fra1* was a direct transcriptional regulatory target of c-Jun and Jun-B in breast cancer cells.

### JNK2 promoted metastasis and p-JNK2 inhibited breast tumor metastasis *in vivo*

**Figure 6:**
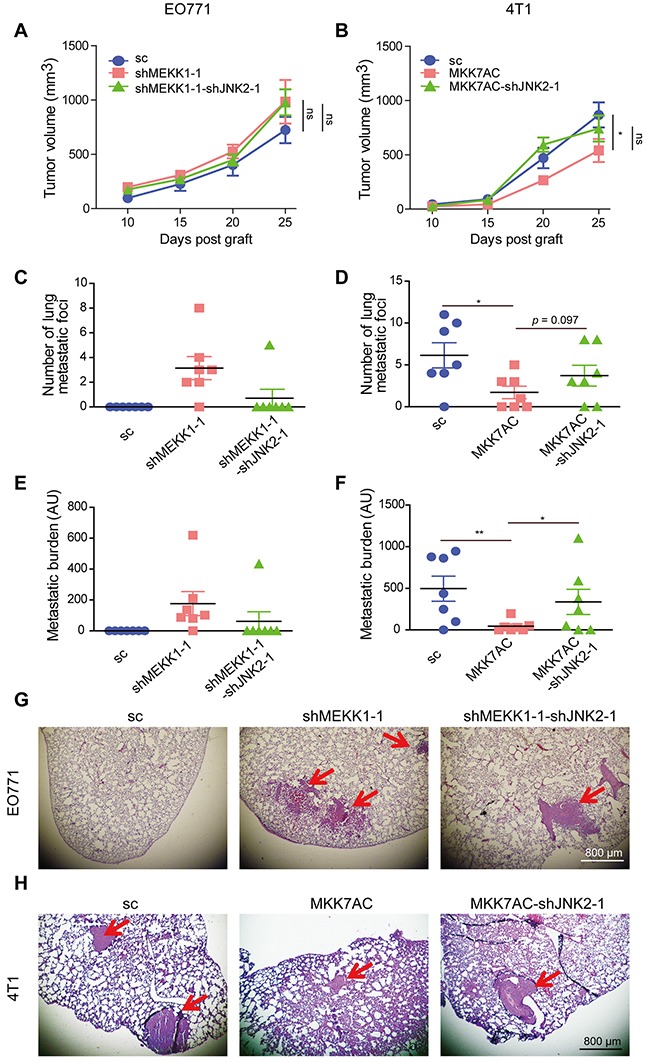
JNK2 promoted metastasis and p-JNK2 suppressed metastasis *in vivo* **(A-B)** Tumor growth curve of EO771 xenografts in C57BL/6 (A) and 4T1 xenografts BALB/c mice (B). Results were presented as mean ± SEM, (n = 7). The tumor volumes at the 25^th^ day  post graft from different groups were compared and analyzed to determine the statistical significance. **(C-D)** Statistical results for the lung metastasis foci number in C57BL/6 (C) and BALB/c mice (D), data were presented as mean ± SEM (n = 7). The significances between different EO771-graft groups were not determined since most of the values in the EO771-sc and EO771-shMEKK1-1-shJNK2-1 groups were zero. Total foci number of lung metastases per animal was counted from all lobes of the lung for each tumor-bearing mouse. **(E-F)** Statistical results for the metastatic burden in tumor-bearing C57BL/6 (E) and BALB/c (F) mice, data were presented as mean ± SEM (n = 7). AU: Arbitrary unit. **(G-H)** Representative H&E staining of lung metastases in tumor-bearing C57BL/6 (G) and BALB/c mice (H). Red arrows indicated the metastatic foci.

To assess the regulatory effect of JNK2 and p-JNK2 on metastasis in *vivo*, EO771 cells were grafted to C57BL/6 mice and 4T1 cells were grafted to BALB/c mice, respectively. No obvious differences for tumor growth were detected among EO771-sc, EO771-shMEKK1-1 and EO771-shMEKK1-1-shJNK2-1 xenografts (Figure 6A). Mice engrafted with EO771-shMEKK1-1 developed substantially more lung metastases (6 of 7 mice) than the EO771-sc xenograft mice (0 of 7 mice) at the day of sacrifice. Likewise, the metastatic foci number and metastatic burden were much heavier (Figure 6C, 6E, 6G). However, mice grafted with EO771-shMEKK1-1-shJNK2-1 exhibited less lung metastasis (1 of 7 mice) relative to the mice engrafted with EO771-shMEKK1-1 (Figure 6C, 6E and 6G).

Inhibited tumor growth was observed in mice engrafted with 4T1-MKK7AC when compared to those engrafted with 4T1-sc. This inhibition was slightly reversed in those grafted with 4T1-MKK7AC-shJNK2-1 (Figure 6B). Most importantly, mice bearing 4T1-MKK7AC cells developed less lung metastasis (4 of 7 mice) relative to those bearing 4T1-sc cells (6 of 7 mice) at the day of sacrifice with significantly reduced metastasis foci number and burden (Figure 6D, 6F, 6H). However, the metastasis-inhibition was reduced in 4T1-MKK7AC-shJNK2-1 xenograft mice (5 of 7 mice. Figure 6D, 6F and 6H). Immunohistochemical staining demonstrated high protein expression level of c-Jun, Jun-B, Fra1, N-cad and low expression of p-JNKs, E-cad in EO771-shMEKK1-1 xenografts as compared to EO771-sc xenografts (Supplementary Figure 4). These results were compromised in EO771-shMEKK1-1-shJNK2-1 xenografts. Compared with 4T1-sc xenografts, the expression of c-Jun, Jun-B, Fra1, N-cad was inhibited, the expression of p-JNKs, E-cad increased in 4T1-MKK7AC xenografts. While these changes were relieved in 4T1-MKK7AC-shJNK2-1 xenografts (Supplementary Figure 4). These results confirmed that the expressions of c-Jun, Jun-B, Fra1 and N-cad in the primary tumor were inversely associated with p-JNK2, while were positively correlated with de-phosphorylated JNK2. The results reinforced the notion that JNK2 promoted metastasis and p-JNK2 inhibited metastasis *in vivo*.

## DISCUSSION

In this study, we investigated the expression of p-JNK and Fra1 in a panel of human as well as murine breast cancer cell lines and found the expression of Fra1 tended to be negatively associated with p-JNKs. Absent/low expression of p-JNKs was detected in most of basal/triple negative breast cancer cells and phosphorylation of JNK2 inhibited cell migration of this types of cells via promoting the ubiquitination of c-Jun and Jun-B.

It is well known that MKK4 and MKK7 are two mainly direct upstream regulators of JNKs. Expression of MKK4 prevented lung metastasis of rat prostate cancer cells in a mouse model [25]. MKK4 mediated metastasis inhibition of ovarian cancer SKOV3ip.1 [26]. MKK7 showed similar protection-effect against metastasis [27]. In this study, overexpression of MKK7AC or MKK4AC increased the expression of p-JNKs in 4T1 and MDA-MB-231 cells, which lead to inhibited expression of Fra1 and decreased cell migration. While these inhibitions were relieved when *JNK2* was silenced and expression of p-JNKs was reduced in 4T1 and MDA-MB-231 cells with *MKK7AC* or *MKK4AC* overexpression. These confirmed that it was p-JNK2, activated by MKK4 and MKK7, contributed to the metastasis inhibition.

However, it should be noted that there was an increase in the migration of 4T1-MKK7AC-shJNK2-1 cells as compared to 4T1-sc cells shown in Figure 3B, 3C, though the Fra1 expression level in the 4T1-sc was slightly higher than that in 4T1-MKK7AC-shJNK2-1 cells (Figure 3A). The slightly increased cell migration with lower expression level of Fra1 was also observed in MDA-MB-231-MKK7AC-shJNK2-1 as compared to MDA-MB-231-sc cells. The underlying mechanism responsible for this phenomenon was complicated. Considering MKK7AC can active both JNK1 and JNK2, the expression of p-JNK1 might increase in 4T1-MKK7AC-shJNK2-1 than in 4T1-sc. Activation of JNK1 promoted the phosphorylation of its downstream proteins to increase cell motility [11, 12], this may be one of the key reasons leading to our observations.

MEKK1 is the upstream regulator of MKK4 and MKK7. In this study, knockdown of *MEKK1* in EO771 cells inhibited the phosphorylation of JNKs, which endowed the cells with higher migration ability and higher expression level of Fra1. Silencing of *JNK2* in EO771-shMEKK1 compromised its promotion effect. This result was consistent with the previous study by Mitra et al. [16]. They found a lower migration potential in polyoma virus middle T antigen (PyVMT) JNK2−/− cells as compared to PyVMT JNK2+/+ cells. The promotion effect of JNK2 on cell migration was also found in another mouse breast cancer cell 4T1.2 [16]. Moreover, when activation of JNK was prevented in the prostate epithelium of ΔMkk4 ΔMkk7 ΔPten mice, rapid development of invasive adenocarcinoma was observed [28].

JNK2 was found to have a 25-fold higher binding affinity for c-Jun than JNK1. This finding led to the notion that JNK2 is the major kinase for c-Jun [29]. Some studies demonstrated the function of JNK2 as a positive regulator of c-Jun [30], whereas other found that JNK2 had a direct negative regulatory role on the expression of c-Jun [6, 31, 32]. In this study, we found that phosphorylated JNK2 promoted the ubiquitination of c-Jun and Jun-B, while de-phosphorylated JNK2 increased the expression of c-Jun and Jun-B. We thus revealed a novel regulatory mechanism of JNK2 on the expression of c-Jun and Jun-B.

**Figure 7:**
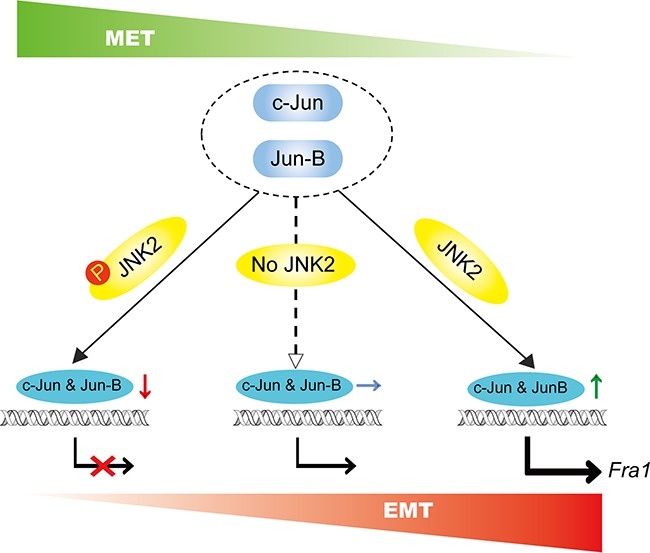
Schematic summary of our proposed model p-JNK2 and JNK2 control the switch between EMT and MET program: p-JNK2 promotes the degradation of c-Jun and Jun-B, which leads to the inhibited expression of Fra1 resulting in the MET process. Conversely, dephosphorylation form of JNK2 promotes the transcript of *Fra1* leading to the promoted EMT.

The EMT program is initially required for invasion and dissemination of tumor cells, whereas MET has been demonstrated to promote colonization and metastatic outgrowth [33]. The expression of Fra1 was revealed to be associated with the aggressive TN breast cancer cells, and depletion of Fra1 led to MET [21]. Our present findings proposed a model revealing that p-JNK2 and JNK2 contributed significantly to the switch between EMT and MET process (Figure 7) by regulating the expression of Fra1 through governing the presence of c-Jun and Jun-B in breast cancer. This study also suggested that when a therapeutic strategy targeting on JNK2 signaling was designed, the phosphorylation status of JNK2 in cancer cells should be considered, since that might affect the therapeutic outcomes for the breast cancer patients even with the similar molecular subtype.

## MATERIALS AND METHODS

### Ethics statement

All animal experiments were performed complied with the guidelines of Nankai University Animal Care and Use Committee. All efforts were made to reduce the suffering of animals during the experiments.

### Cell culture and DNA constructs

ZR-75-1, BT-474, T-47D, MDA-MB-231, MDA-MB-453, MDA-MB-436, MDA-MB-435 and HCC1937 cells were obtained from the Cell Bank of Chinese Academy of Sciences (Shanghai, China). MCF7, 4T1, 4TO7 and EO771 cells were obtained from Dr. Ralph A. Reisfeld (The Scripps Research Institute, CA, USA). EMT6 was obtained from the Laboratory Animal Research Center of the Fourth Military Medical University (Xi’an, Shanxi, China).

shRNAs with the following sequences in Supplementary Table 1 were utilized. The targeting shRNAs were cloned into the pLv-H1-EF1α-puro or pLv-H1-EF1α-Bsd plasmid (Biosettia Inc., San Diego, CA, USA). Lentivirus infections were performed following the manufactures’ instructions. Stable polyclonal cell lines were selected by adding puromycin or blasticidin to the cell culture medium.

### RNA isolation, reverse transcription (RT) and real-time quantitative PCR analysis

Cells were washed twice in ice-cold PBS. Total RNA was isolated by using TRIzol and complementary DNA was synthesized using oligo-(dT) primers. Samples were analyzed by real-time quantitative PCR with the Bio-Rad CFX96 Light Cycler using the TransStart® Top Green qPCR SuperMix (cat.# AQ131-02, TransGen Biotech, Beijing, China, PR) and the primer sequences were summarized in Supplementary Table 2. Results were normalized to *Gapdh*. The 2^−ΔΔCt^ method was used to determine relative fold-changes for the expression of mRNA. The experiments were performed in triplicate.

### Western blotting

Different cell lysates were developed with RIPA buffer in the presence of Phosphatase Inhibitor Cocktail (PIC), PIC 2 and PIC 3 (cat. # P8340, P5726 and P0044, Sigma-Aldrich, St Louis, MO, USA). Protein (30 μg) was loaded onto 5–12% Tris-acrylamide gels and blotted with antibodies including: anti-β-actin, phospho-JNK (Thr183/Tyr185), JNK2, MKK7, MKK4, c-Jun, Jun-B, phospho-c-Jun (Ser73), phospho-c-Jun (Ser63) (cat. # 4970, 4668, 9258, 4172, 9152, 9165, 3753, 3270, 9261, Cell Signaling Technology, Danvers, MA, USA), anti-MEKK1, (cat. # ab55653, abcam, Cambridge, UK), anti-Fra1, Ub and JunD (cat. # sc-28310, sc-8017, sc-271938, Santa Cruz Biotechnology, Inc., Dallas, Texas, USA), anti-Cytokeratin 14 and Cytokeratin 8 (cat. # 10143-1-AP, 10384-1-AP, Proteintech, Wuhan, China), anti-N-cadherin (cat. # 610920, BD Bioscience, San Jose. CA, USA), These primary antibodies were detected with proper secondary antibodies. Proteins were detected by ECL detection reagent (cat. #WBKLS0500, Millipore, Billerica, MA, USA).

### Transwell and wound healing assay

These experiments were performed and the wound closure rate were calculated following the procedure described before [34]. Specifically, for the transwell assay, the cells that penetrated and attached to the bottom of the filter of the transwell were staining of 0.1% crystal violet followed by microscope imaging and counted as cell number per image field (cell number/field). They were then dissolve in 50% acetic acid, the intensity of crystal violet were measured as absorbance at 570nm (OD_570_).

### Immunofluorescence staining

Cell cultures were fixed in 4% parafor-maldehyde followed by blocking with 1% bovine serum albumin in PBS. The following antibodies were used for immunofluorescence: anti-E-cadherin and N-cadherin (cat. # 610182, 610920, BD Bioscience, San Jose, CA, USA), anti-Cytokeratin 14 and Cytokeratin 8 (cat. # 10143-1-AP, 10384-1-AP, Proteintech, Wuhan, China). Cells were incubated with the above primary antibodies, followed by incubation with the appropriate secondary antibodies conjugated with Alexa Fluor-488, or -594 (cat. # ab150077, ab150080, ab150113, ab150116, abcam, Cambridge, UK).

### Chromatin immunoprecipitation

The protein-DNA complexes were cross-linked by incubating the cells with Formaldehyde. Using sonication, the chromatin of samples were sheared to an average length of 400–600 bp. Samples were immunoprecipitated using anti-c-Jun and anti-Jun-B antibody. The enriched DNA was then de-cross-linked and analyzed by quantitative PCR with following primers in Supplementary Table 3. Relative occupancy values were calculated by determining ratios of the amount of immunoprecipitated DNA to that of the input sample.

### Co-immunoprecipitation (Co-IP)

The cultured cells were washed with pre-chilled PBS for 2 times. Cell lysates were harvested by adding 1 mL of RIPA buffer in the presence of PIC, PIC 2 and PIC 3 to 10-cm plate of cells followed by scrapping. Cell lysate was pre-cleared with 20 μL suspended Protein G agarose (cat # 20399, Thermo Fisher Scientific). The mixture was rotated at 4°C for 30 min to 1 hour (h) and was then spun at 1,000 g for 1 min at 4°C. The supernatant was transferred into a clean microcentrifuge tube and incubated with primary antibody at 4°C with continuous rotation for overnight. 20 μL of Protein G agarose was added to and incubated with the samples at 4°C with rotation for 1-2 h. The agarose beads were spun down at 1,000 g for 1 min at 4°C. The supernatant was discarded carefully to avoid the disturbance of the beads. The beads were washed with PBS for 3 times. After the last wash, the buffer was removed as much as possible. 20-50 μL of loading buffer was added and mixed to re-suspend the beads. The samples were incubated with loading buffer for 10 min at 95°C, and then subject to SDS-PAGE analysis.

For the ubiquitination assay, the cells were treated with 10 μM MG132 (cat. # M8699, Sigma-Aldrich, St Louis, MO, USA) for 4-6 h prior to harvest to enrich the ubiquitination signal.

### Promoter-luciferase reporters

*Fra1* promoter luciferase plasmids were constructed as F1-Luc described in Figure 5I. To perform the promoter-luciferase assays, cells were seeded in 24-well culture plates 24 h before transfection. Then the cells were co-transfected with promoter-luciferase and SV40-Renilla-luciferase (pRL-SV40). Cells were transfected with 100 ng of promoter-luciferase and 30 ng of pRL-SV40. The luciferase activity was measured at 24 h after transfection by using the Dual-Luciferase® Reporter Assay System (cat. # E1910, Promega). The data were expressed as the ratio of firefly-luciferase (FL) reading versus renilla-luciferase (RL) reading (FL/RL).

### Xenograft assays

Six-week-old female BALB/c mice were each implanted subcutaneously around the fourth mammary gland with 1×10^5^ 4T1-sc, 4T1-MKK7AC, 4T1-MKK7AC-shJNK2-1 cells, respectively. Six-week-old C57BL/6 mice were each implanted subcutaneously around the fourth mammary gland with 3×10^5^ EO771-sc, EO771-shMEKK1-1, EO771-shMEKK1-1-shJNK2-1 tumor cells, respectively. Tumor size was measured by a caliper, and tumor volume was calculated using the formula: ½ (Length×Width^2^). The mice were sacrificed and five lobes of lung for each mouse of each group were harvested after 30 days post graft. The lung lobes were fixed and then stained with hematoxylin and eosin (H&E) for analysis of tumor metastasis.

### Immunohistochemistry

Immunostaining was performed on xenograft primary tumors. JNK2, p-JNK, c-Jun, Jun-B, Fra1, E-cadherin, N-cadherin were stained by using the primary antibodies described above. Standard streptavidin–biotin method was applied and 3,3′-Diaminobenzidine (DAB) substrate was used. The images were recorded by Olympus BX51 Epi-fluorescent microscopy under a 40× objective (Olympus Co., Tokyo, Japan).

### Statistical analyses

*t*-test was used to determine the significance. All data were presented as mean + standard error of the mean (SEM) unless otherwise specified. A value of *p*<0.05 was used as the criterion for statistical significance. “*” indicates *p*<0.05, “**” indicates *p*<0.01, “ns” indicates not significant.

## SUPPLEMENTARY MATERIALS FIGURES AND TABLES


