# Supplementary material for: Alternative promotion and suppression of metastasis by JNK2 governed by its phosphorylation
Source: Oncotarget. 2017 Apr 28;8(34):56569–81. doi: 10.18632/oncotarget.17507 (PMC5593584; doi:10.18632/oncotarget.17507)
Supplement: Supplementary file 1 [file oncotarget-08-56569-s001.pdf]

Alternative promotion and suppression of metastasis by JNK2 governed by its phosphorylation

SUPPLEMENTARY FIGURES AND TABLES

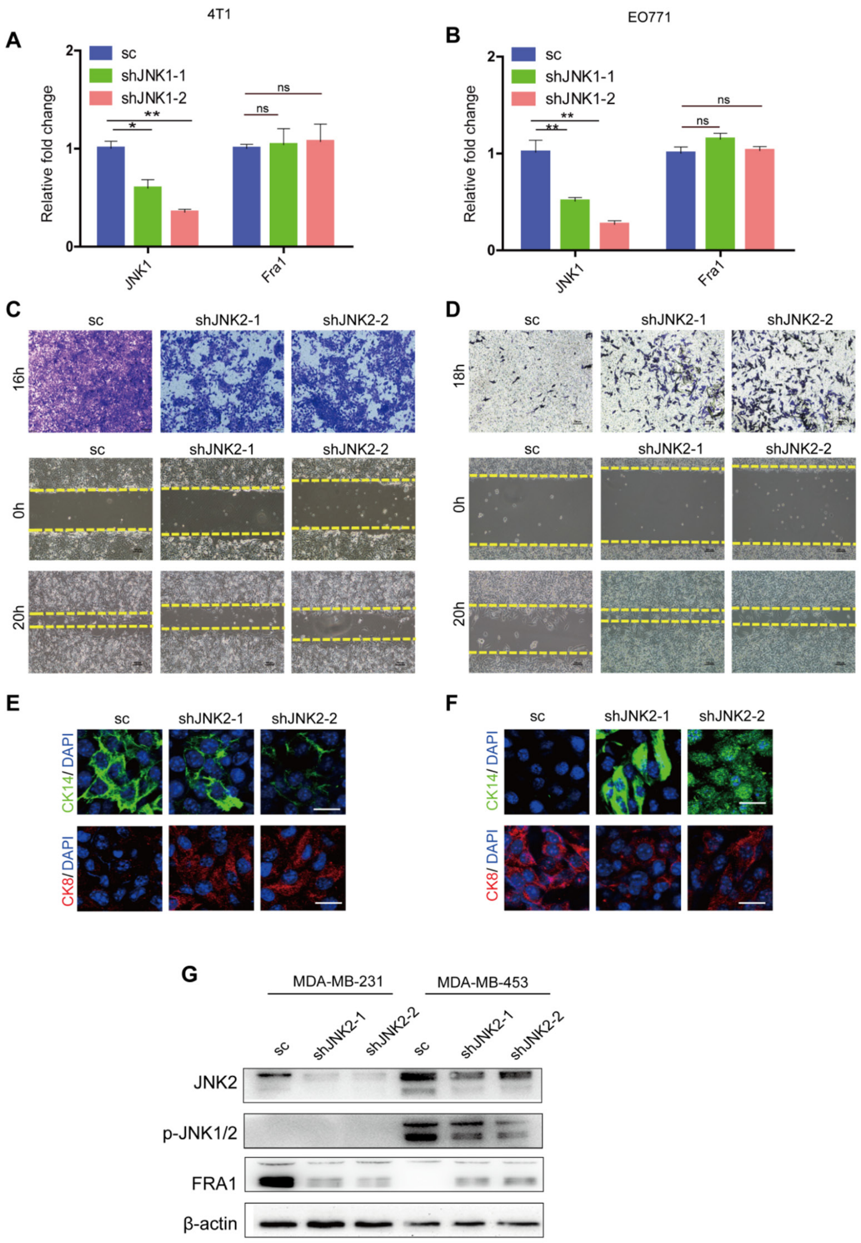

Supplementary Figure 1: Down-regulation of *JNK2* showed opposite effects on cell migration in different breast cancer cells. (A-B) Quantitative RT-PCR for mRNA-expression changes of *JNK1* and *Fra1* genes in 4T1 cells (A) and EO771 cells (B) following *JNK1* knockdown, (n=3). **(C-D)** Cell migration and cell mobility assay of 4T1 (C) and EO771 (D) infected with lentivirus carrying shRNAs targeting JNK2 or the non-targeting shRNA control (sc) measured by transwell (upper panel) and wound healing (lower panel). The edge of the wound was indicated by the dash line. **(E-F)** Immunofluorescent staining of CK8 (red), CK14 (green) in 4T1 cells (E) and EO771 cells (F), the nuclei were counterstained with DAPI (blue). Scale bar: 50 µm. **(G)** Western blot analysis of JNK2, p-JNK1/2 and FRA1 in MDA-MB-231 cells and MDA-MB-453 cells infected with lentivirus carrying shRNAs targeting JNK2 or the non-targeting shRNA control (sc).

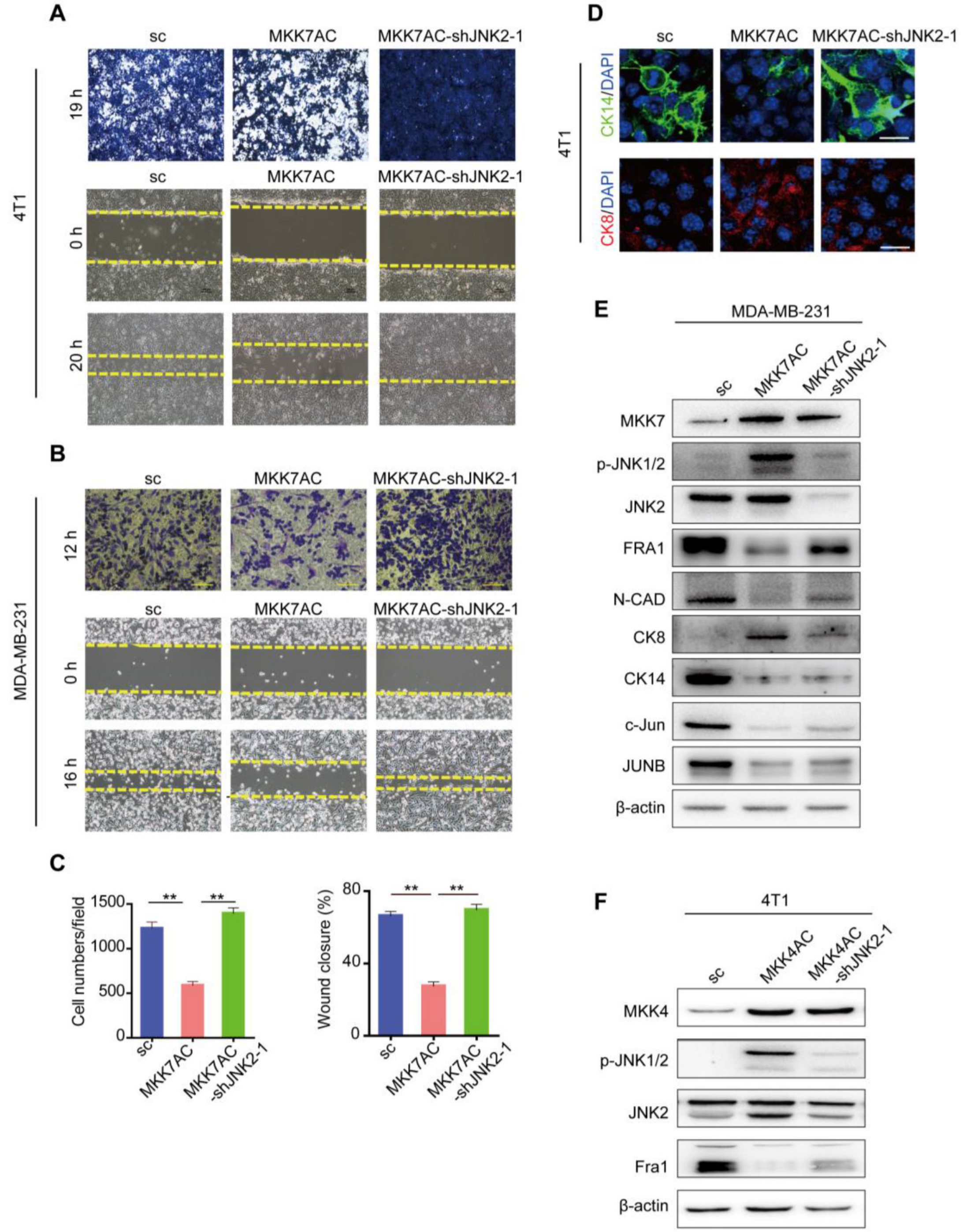

Supplementary Figure 2: Overexpression of *MKK7AC* promoted the phosphorylation of JNK2 and inhibited the migration of 4T1 and MDA-MB-231 cells. **(A-B)** Cell migration and motility of 4T1 cells (A) and MDA-MB-231 (B) were detected by transwell (upper panel) and wound healing (lower panel). The edge of the wound was indicated by the dash line. **(C)** Statistical results for the transwell assay (left panel, n = 5) and wound healing assay (right panel, n = 8) performed in MDA-MB-231 cells. **(D)** Immunofluorescent staining of CK8 (red), CK14 (green) in 4T1 cells, the nuclei were counterstained with DAPI (blue). Scale bar: 50 μm. **(E)** Western blot results of MKK7, p-JNK1/2, JNK2, FRA1, N-CAD, CK8, CK14, c-Jun and JUNB in MDA-MB-231 cells. **(F)** Western blot results of MKK4, p-JNK1/2, JNK2 and Fra1 in 4T1 cells.

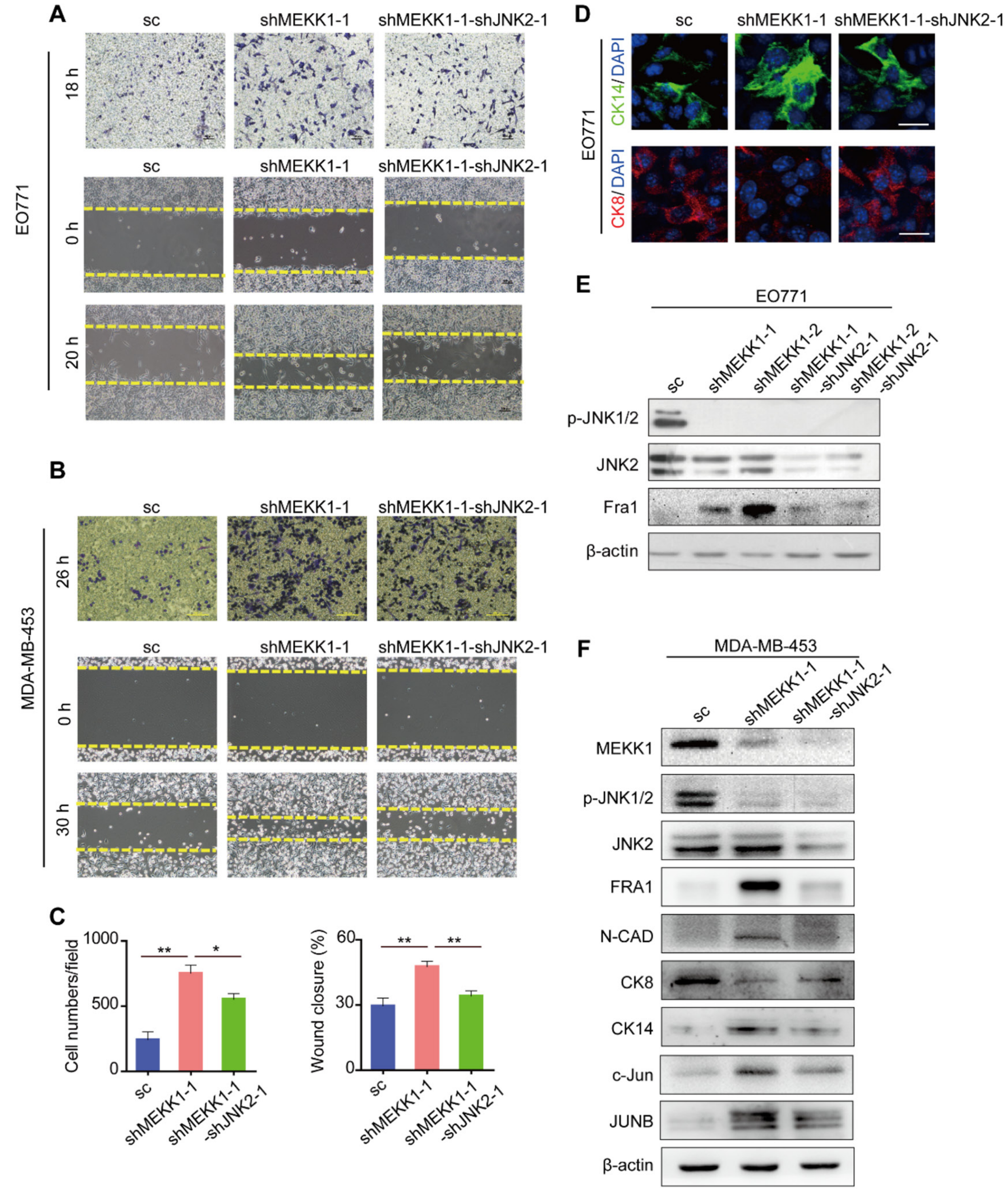

Supplementary Figure 3: Silencing of *MEKK1* inhibited the phosphorylation of JNK2 and promoted cell migration in EO771 and MDA-MB-453 cells. **(A-B)** Cell migration and motility of EO771 cells (A) and MDA-MB-453 cells (B) were detected by transwell (upper panel) and wound healing (low panel). The edge of the wound was indicated by the dash line. **(C)** Statistical results for the transwell assay (left panel, n = 3) and wound healing assay (right panel, n = 7) performed in MDA-MB-453 cells. **(D)** Immunofluorescent staining of CK8 (red), CK14 (green) in EO771 cells, the nuclei were counterstained with DAPI (blue). Scale bar: 50 µm. **(E)** Western blot results of p-JNK1/2, JNK2 and Fra1 in EO771 cells. **(F)** Western blot results of MEKK1, p-JNK1/2, JNK2, FRA1, N-CAD, CK8, CK14, c-Jun and JUNB in MDA-MB-453 cells.

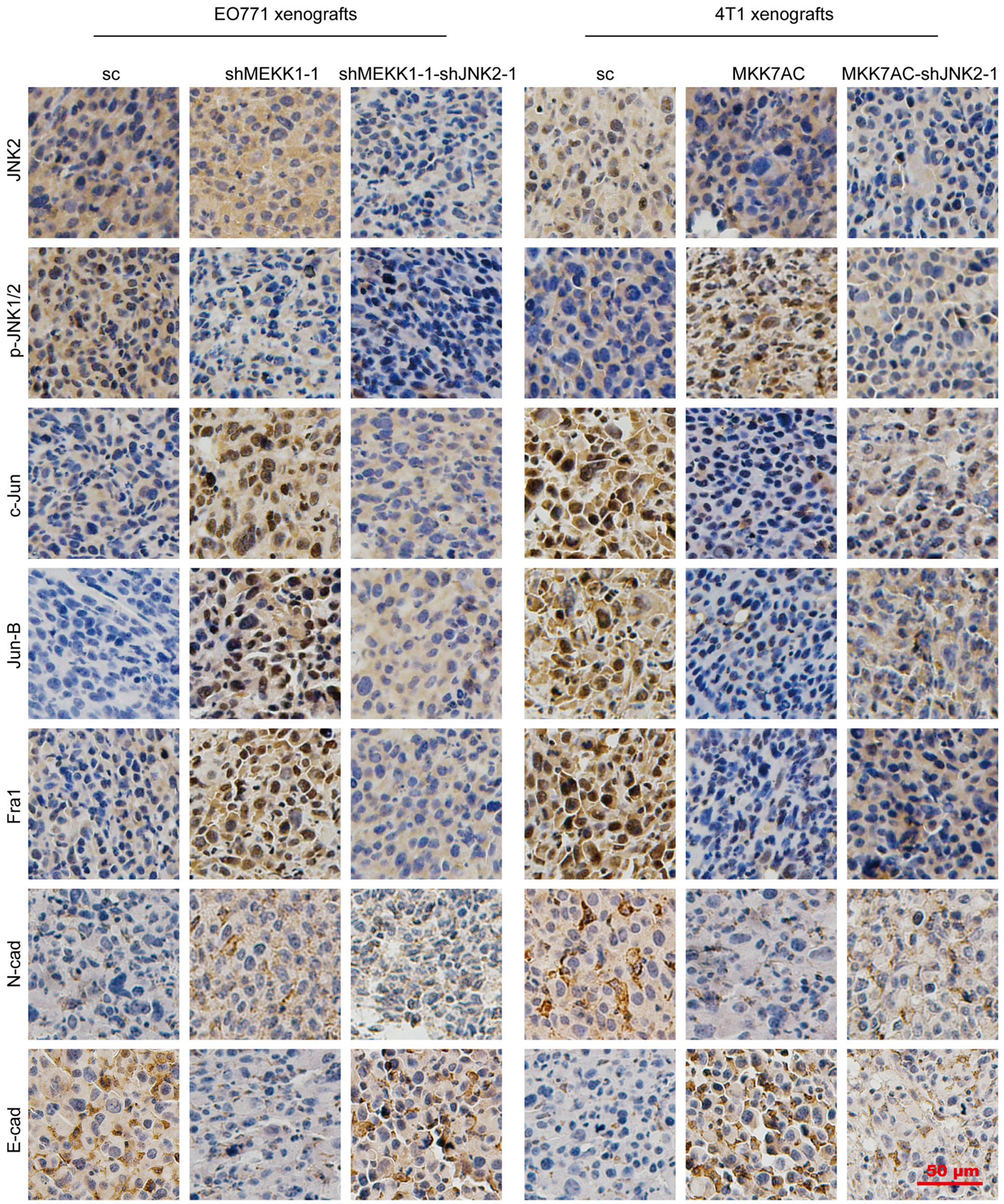

Supplementary Figure 4: Representative immunohistochemistry staining of JNK2, p-JNK1/2, c-Jun, Jun-B, Fra1, N-cad and E-cad in primary EO771 xenografts from C57BL/6 mice and 4T1 xenografts from BALB/c mice.

Supplementary Table 1: shRNAs with the following sequences were utilized in this study

shRNAs	Sequence
shJNK2-1 (Mus)	AAAAGGAGCCCAAGGAATTGTTTGTTTGGATCCAAACAAACAATTCCTTGGGCTCC
shJNK2-2 (Mus)	AAAAGCATTCAGCTGGTATCATTCATTGGATCCAATGAATGATACCAGCTGAATGC
shJNK1-1 (Mus)	AAAAGCAGAAGCAAACGTGACAACATTGGATCCAATGTTGTCACGTTTGCTTCTGC
shJNK1-2 (Mus)	AAAAGCTCAGGAGCTCAAGGAATAGTTGGATCCAACTATTCCTTGAGCTCCTGAGC
shJNK2-1 (Homo)	AAAAGGGATTGTTTGTGCTGCATTTTTGGATCCAAAAATGCAGCACAAACAATCCC
shJNK2-2 (Homo)	AAA AGCCTTCAGATGCAGCAGTAAGTTGGATCCAACTTACTGCTGCATCTGAAGGC
shMEKK1-1 (Mus/Homo)	AAAAGCTGCTTATTCTCTAGAAACTTTGGATCCAAAGTTTCTAGAGAATAAGCAGC
shMEKK1-2 (Mus)	AAAAGGTGTCGATTACGTCTTAAGTTTGGATCCAAACTTAAGACGTAATCGACACC
shc-Jun-1 (Mus)	AAAAGCAAAGATGGAAACGACCTTCTTGGATCCAAGAAGGTCGTTTCCATCTTTGC
shc-Jun-2 (Mus)	AAAAGCTACAGTACCCCTAAGATCCTTGGATCCAAGGATCTTAGGGTTACTGTAGC
shJunb-1 (Mus)	AAAAGGATCAGACACAGGCGCATCTTTGGATCCAAAGATGCGCTGTGTCTGATCC
shJunb-2 (Mus)	AAAAGCATCTCTGAAGCTAGCCTCCTTGGATCCAAGGAGGCTAGCTTCAGAGATGC

Supplementary Table 2: The following primers were used in real-time quantitative RT-PCR

Gene name	Primers
JNK2 (*Mus*)	Forward: 5’-GTGACAGTAAAAGCGATGG-3’
	Reverse: 5’-TGGTTCTGAAAAGGACGAC-3’
Fra1 (*Mus*)	Forward: 5’-GGATGGTGCAGCCTCATTTCC-3’
	Reverse: 5’-GCTAGCTTGTTCCGCTCGCGT-3’
Ecad (*Mus*)	Forward: 5’-GGGACAAAGAAACAAAGGTT-3’
	Reverse: 5’-TGACACGGCATGAGAATAGA-3’
Ncad (*Mus*)	Forward: 5’-CCCCCAAGTCCAACATTTC-3’
	Reverse: 5’-CCGCCGTTTCATCCATACC-3’
Fn1 (*Mus*)	Forward: 5’-CATTCCACCTTACAACACC-3’
	Reverse: 5’-AGTCAAGCCAGACACAACA-3’
Mmp2 (*Mus*)	Forward: 5’-GTACTGGGTCTATTCTGCTA-3’
	Reverse: 5’-TGTATGTCTTCTTGTTCTTA-3’
Mmp9 (*Mus*)	Forward: 5’-CCCTGTGTGTTCCCGTTCAT-3’
	Reverse: 5’-GGTCATAGTTGGCTGTGGTG-3’
Vim (*Mus*)	Forward: 5’-AGCCTCTATTCCTCATCCC-3’
	Reverse: 5’-TGCAGTTCTACCTTCTCGT-3’
uPA (*Mus*)	Forward: 5’-CTCCCTCCTTTAAATGTGGT-3’
	Reverse: 5’-CTTCCCTGTAGTATTCGTGC-3’
Slug (*Mus*)	Forward: 5’-AGAGATCCTCACCTCGGGA-3’
	Reverse: 5’-GAAGGCGGGGGACTTACAC-3’
Snail (*Mus*)	Forward: 5’-CCGATGAGGACAGTGGCAAA-3’
	Reverse: 5’-CCAGGGAAGGCGATGAAGGC-3’
Twist (*Mus*)	Forward: 5’-GCGCAAGATCATCCCCACGC-3’
	Reverse: 5’-GCCCCCTCCATCCTCCAGAC-3’
Zeb1 (*Mus*)	Forward: 5’-CAGGGTTACTCTTGTGTGG-3’
	Reverse: 5’-CTTTTGTGGTTCCTTTTTT-3’
c-Jun (*Mus*)	Forward: 5’-GCCGCCCCTGTCCCCTATC-3’
	Reverse: 5’-CTGTGCCACCTGTTCCCTG-3’
Junb (*Mus*)	Forward: 5’-TTGCGGACGGTTTTGTCAAA-3’
	Reverse: 5’-AGGGTGCAGAGGCTGGGGAG-3’
JNK1 (*Mus*)	Forward: 5’-CAAGGAATAGTGTGTGCA-3’
	Reverse: 5’-TAGGGATTTCTGTGGTGT-3’
Gapdh (*Mus*)	Forward: 5’-ACCACAGTCCATGCCATCAC-3’
	Reverse: 5’-TCCACCACCCTGTTGCTGTA-3’

Supplementary Table 3: The following primer sequences were used in quantitative PCR for analyzing the occupancy of c-Jun and Jun-B on the *Fra1* genomic loci

Position	Primers
P1	Forward: 5’-CAGTGCCCGCCCAGGTCTT-3’
	Reverse: 5’-TCCGCTCCTCTGCTCCAAA-3’
P2	Forward: 5’-GGAGCAGAGGAGCGGAGAT-3’
	Reverse: 5’-CAAGTTTGGGCTGAGGTGG-3’
P3	Forward: 5’-CCCCGTGGTGCAAGTGGTTC-3’
	Reverse: 5’-TGGGCTGGATGTTCGGTAGG-3’
P4	Forward: 5’-GAGGGAAGGAGCTGCGTC-3’
	Reverse: 5’-CCCCGTTCTGGGCTAGAA-3’
P5	Forward: 5’-TATCCCCAGTACAGTCCC-3’
	Reverse: 5’-AAATTCCCTCGTAGCAAG-3’
